# Comprehensive transcript-level analysis reveals transcriptional reprogramming during the progression of Alzheimer’s disease

**DOI:** 10.3389/fnagi.2023.1191680

**Published:** 2023-06-15

**Authors:** Hao Wu, Jiao Wang, Xiaoyuan Hu, Cheng Zhuang, Jianxin Zhou, Peiru Wu, Shengli Li, Robert Chunhua Zhao

**Affiliations:** ^1^Laboratory of Molecular Neural Biology, School of Life Sciences, Shanghai University, Shanghai, China; ^2^H. Milton Stewart School of Industrial and Systems Engineering, College of Engineering, Geogia Institute of Technology, Atlanta, GA, United States; ^3^Precision Research Center for Refractory Diseases, Shanghai General Hospital, Institute for Clinical Research, Shanghai Jiao Tong University School of Medicine, Shanghai, China; ^4^School of Basic Medicine, Peking Union Medical College, Institute of Basic Medical Sciences Chinese Academy of Medical Sciences, Beijing, China

**Keywords:** transcript expression, alternative splicing, Alzheimer’s disease, isoform switch, RNA binding proteins, asymptomatic AD

## Abstract

**Background:**

Alzheimer’s disease (AD) is a common neurodegenerative disorder that has a multi-step disease progression. Differences between moderate and advanced stages of AD have not yet been fully characterized.

**Materials and methods:**

Herein, we performed a transcript-resolution analysis in 454 AD-related samples, including 145 non-demented control, 140 asymptomatic AD (AsymAD), and 169 AD samples. We comparatively characterized the transcriptome dysregulation in AsymAD and AD samples at transcript level.

**Results:**

We identified 4,056 and 1,200 differentially spliced alternative splicing events (ASEs) that might play roles in the disease progression of AsymAD and AD, respectively. Our further analysis revealed 287 and 222 isoform switching events in AsymAD and AD, respectively. In particular, a total of 163 and 119 transcripts showed increased usage, while 124 and 103 transcripts exhibited decreased usage in AsymAD and AD, respectively. For example, gene *APOA2* showed no expression changes between AD and non-demented control samples, but expressed higher proportion of transcript *ENST00000367990.3* and lower proportion of transcript *ENST00000463812.1* in AD compared to non-demented control samples. Furthermore, we constructed RNA binding protein (RBP)-ASE regulatory networks to reveal potential RBP-mediated isoform switch in AsymAD and AD.

**Conclusion:**

In summary, our study provided transcript-resolution insights into the transcriptome disturbance of AsymAD and AD, which will promote the discovery of early diagnosis biomarkers and the development of new therapeutic strategies for patients with AD.

## Introduction

Alzheimer’s disease (AD) is one of the most common neurodegenerative disorders around the whole world (Querfurth and LaFerla, 2010; No authors listed, 2023), which is primarily characterized by symptoms such as memory loss, cognitive decline, and behavioral changes (Ashrafian et al., 2021). The presence of β-amyloid (Aβ)-containing plaques and tau-containing neurofibrillary tangles is very prevalent in the brain of patients diagnosed with AD (Knopman et al., 2021). Molecular mechanisms underlying AD are complex and have not been fully understood, but recent studies have highlighted roles of transcriptome dysregulation in the development and progression of this disease (Biamonti et al., 2021). Asymptomatic AD (AsymAD) was considered as the asymptomatic stage of AD based on the NIA research framework (Jack et al., 2018). Of note, older adults with AsymAD have autopsy-confirmed AD pathology but not cognitive impairments (Hohman et al., 2016). The duration of AsymAD can vary among individuals but typically lasts 6–10 years, depending on the age of disease onset (Porsteinsson et al., 2021). A better understanding of the long asymptomatic stage of AD enables the development of intervention and secondary prevention strategies for asymptomatic individuals at risk, which prevent the development of significant irreversible neuronal dysfunction and loss (Crous-Bou et al., 2017). Strategies that combine high-throughput RNA sequencing (RNA-seq) data and computational algorithms have facilitated transcriptome-wide investigation of human complex diseases at an unparalleled scale and resolution (Li et al., 2019; Zhao, 2019; Hu et al., 2022), which has promoted the discovery of diagnosis and therapy targets for various diseases.

Alternative splicing (AS) is one of the most important post-transcriptional regulations that contribute to transcriptome diversity (Wang et al., 2008; Djebali et al., 2012), which is a process that different RNA transcripts are generated from a single gene and might lead to the production of functionally diverse protein isoforms (Keren et al., 2010). Aberrant AS has been implicated in a wide range of human diseases, including AD (Koch, 2018; Biamonti et al., 2021). Specifically, dysregulation of AS has been shown to affect the expression of genes involved in key processes such as synaptic function, neuroinflammation, and tau protein metabolism, which are all critical components of AD pathology (Ishunina, 2021). Despite numerous studies investigating the role of transcriptome in AD, a comprehensive analysis of transcript-level alterations and their biological and clinical implications in AD has been lacking. RNA binding proteins (RBPs) are major regulators of AS, which bind to specific regions of precursor RNAs to modulate splicing efficiency and the selection of splice sites during AS process (Gerstberger et al., 2014; Xiao et al., 2019). The regulation of RBPs on dysregulated AS has been investigated in many human diseases (Li et al., 2019; Hu et al., 2020; Liu and Cao, 2023). However, the dysregulation of this splicing machinery in AD is unclear.

In order to comprehensively characterize the transcriptome dysregulation in AD, we collected RNA-seq datasets of a large AD cohort. Our analysis revealed the dysregulation of AD transcriptome at transcript level, and examined AS events that might cause transcript variations. We further investigated RBP-ASE regulatory networks and highlighted RBPs that might play important regulatory roles in transcriptome dysregulation in AsymAD or AD. These analyses were comparatively performed in AsymAD and AD, which highlighted the transcriptome dysregulation that were different between these two disease stages.

## Materials and methods

### RNA-seq datasets of Alzheimer’s diseases

Bulk RNA-seq expression profiles of genes and transcripts were retrieved from the Religious Orders Study/Memory and Aging Project (ROSMAP) (Raj et al., 2018). A total of 454 brain samples, including 145 non-demented control samples and 309 disease samples. The disease samples were further divided into AsymAD (*n* = 140) and AD samples (*n* = 169). AsymAD was considered as the early stage of AD (Jack et al., 2018). AD samples were from sporadic AD cohorts (Haure-Mirande et al., 2019). Postmortem neuropathological evaluation of neuritic plaque distribution was performed according to the CERAD score which is a semiquantitative measure of neuritic plaques, and the severity of neurofibrillary tangle (NFT) pathology was assessed with the Braak staging system. Other neuropathological diagnoses were made in accordance with established criteria and guidelines. Sample classification harmonization was performed according to the previous study (Johnson et al., 2020). Particularly, samples with CERAD score 3–4 and Braak 0–3 without dementia at last evaluation were defined as non-demented control; samples with CERAD score 1–3 and Braak 3–6 without dementia at last evaluation were defined as AsymAD; samples with CERAD 1–2 and Braak 3–6 with dementia at last evaluation were defined as AD. Dementia was defined as MMSE < 24. These datasets were downloaded from the AMP-AD Knowledge Portal.^1^ The expression profiles were normalized to control different sequencing strategies and sequencing depths in values of transcript per million mapped reads (TPM). Both gene and transcript expression profiles were filtered by removing low-expression genes or transcripts. Specifically, transcripts or genes with expression level >0.1 TPM in no less than 5% of all samples were kept for further analysis.

### Differentially expressed transcript analysis

The differentially expressed transcript (DET) analysis was performed between the AsymAD and non-demented control, AD and non-demented control samples, respectively. The Wilcoxon’s rank-sum test was employed in the difference evaluation. A transcript with fold change >1.5 or < 0.67 and FDR value <0.05 was considered as DET in AsymAD or AD samples. Protein-coding genes with DETs were adopted to perform functional enrichment analysis by using the clusterProfiler R package (version 4.1.4) (Wu et al., 2021). Gene lists of biological processes were derived from the Molecular Signature Database (MSigDB) (Liberzon et al., 2015).

### Expression correlation analysis of transcripts and host genes

For each transcript, a Pearson correlation coefficient was computed with the corresponding host gene by using normalized expression values across all samples. Host genes are those genes that the corresponding transcripts are transcribed from, and transcript-host gene pairs were extracted from the GENCODE annotation (Frankish et al., 2019). The expression correlation between a transcript and host gene was considered positive if their Pearson correlation coefficient was higher than 0.3, negative if their correlation coefficient was lower than -0.3. The threshold of *p*-values was set at 0.05. Otherwise, the expression of a transcript was considered as irrelevant with its host gene.

### Identification of alternative splicing events

The alternative splicing events (ASEs) were identified by using the SUPPA2 (version 2.3) software (Trincado et al., 2018). Briefly, ASEs were inferred from the comparison of transcript structures and expression levels in the same genes. The value of percent spliced in (PSI) was calculated for each ASE in each sample (Li et al., 2019). ASEs were further processed to generate high-confidence events by retaining events with a PSI value greater than 0.1 in no less than 5% of samples. To detect differentially spliced ASEs, we used the Wilcoxon singed-rank test to compare the PSI values between non-demented control and AsymAD or AD samples. ASEs with a median PSI > 0.1 and a corrected *p*-value < 0.05 were considered differentially spliced.

### Identification of isoform switching events

Isoform switching events of individual genes were identified in each sample. Briefly, summed expression of each gene was calculated from all transcripts (isoforms) that belong to corresponding host genes. The isoform fraction (IF) of each isoform was calculated as follows:


$$I\,F_{i}=\frac{T_{i}}{\sum_{j=1}^{n}E_{j}}$$

where *IF*_*i*_ represents the IF of isoform *i*, *T_i_* is the expression level of isoform *i*, *E_j_* denotes the expression level of isoforms in gene *j*, *n* indicates the number of isoforms in gene *j*.

The significance of the change in isoform usages between AD and non-demented control, or AsymAD and non-demented control group was evaluated by Student’s *t*-test. The difference in IF of each isoform was calculated as follows:


$$d\,I\,F_{i}=\frac{\sum_{k=1}^{n}I\,F_{k}}{n}-\frac{\sum_{j=1}^{m}I\,F_{j}}{m}$$

where *dIF*_*i*_ is the difference in IF of isoform *i*, *IF*_*i*_ denotes the IF value of isoform *i* in group *k*, *n* is the sample number of group *k*, *IF*_*j*_ denotes the IF value of isoform *i* in group *j*, *m* is the sample number of group *j*.

A gene that has at least one isoform with | dIF| > 0.1 and FDR < 0.05 was considered to have isoform switching events in AsymAD or AD samples.

### Construction of RBP-ASE regulatory network

A list of RBPs were retrieved from the Encyclopedia of RNA Interactomes (ENCORI) database (Li et al., 2014). ENCORI database also provided RBP binding regions that were generated from the integration of large-scale high-throughput cross-linking immunoprecipitation (CLIP) experiments. Spearman correlations were calculated between each RBP and differentially spliced ASE. In the correlation calculation, we only considered RBP-ASE pairs that RBPs had binding evidence of CLIP signals within regions +/-300 bp around the corresponding splicing sites. RBPs that showed no less than 10% expression changes between non-demented control samples and AsymAD or AD samples were considered in the correlation calculation. RBP-ASE pairs that had a Spearman correlation >0.5 or <-0.5 and *p*-value < 0.05 were considered significantly correlated.

### Statistical analysis

Statistical analysis and data visualization in this study were performed by using the R software (R Foundation for Statistical Computing, Vienna, Austria).^2^ Unless specifically stated, all tests were two-sided, and p or FDR values <0.05 were considered statistically significant.

## Results

### A transcript-level resolution atlas of AD transcriptome

**FIGURE 1:**
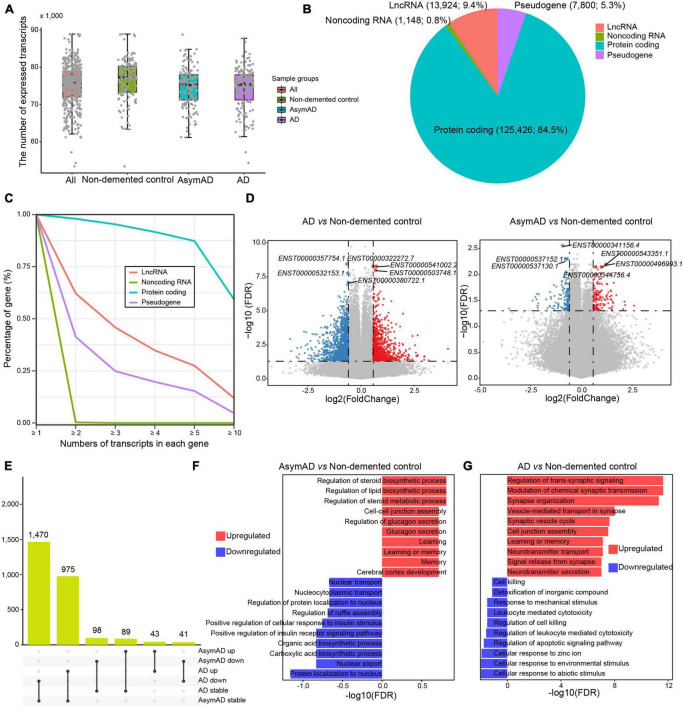
The expression landscape of transcripts in Alzheimer’s disease (AD). **(A)** The number of transcripts expressed in different sample groups. **(B)** Pie chart showing the percentages of different transcript types. **(C)** The percentage distribution of genes with different transcript numbers. **(D)** Volcano plots showing the expression difference of transcripts in AD (left panel) or asymptomatic AD (AsymAD) (right panel) samples. **(E)** Upset chart showing the overlaps of differentially expressed transcripts between AD and AsymAD samples. **(F)** Bar plots showing enriched biological processes by upregulated and downregulated transcripts in AsymAD samples, respectively. **(G)** Bar plots showing enriched biological processes by upregulated and downregulated transcripts in AD samples, respectively.

To characterize a high-resolution dysregulation in AD transcriptome, we performed transcript-level investigation in 454 AD-related samples. In terms of median value, 77,258, 75,253, and 75,355 transcripts were detected in the non-demented control, AsymAD, and AD samples, respectively (Figure 1A). The vast majority (*n* = 125,426, 84.5%) of these expressed transcripts were derived from protein-coding genes, while approximately 10 percent (*n* = 13,924, 9.4%) were from long non-coding RNA (lncRNA) genes and 5.3% of transcripts were from pseudogenes (Figure 1B). Most genes expressed multiple transcripts, especially protein coding genes, wherein over 80% of protein coding genes expressed more than five transcripts (Figure 1C). More than 60% of lncRNA genes expressed no less than two transcripts, while about one third pseudogenes expressed at least two transcripts. We next identified DETs in AsymAD and AD samples, respectively. A total of 1,039 upregulated transcripts and 1,545 downregulated transcripts were identified in AD samples compared to non-demented control samples (Figure 1D and Supplementary Table 1). In AsymAD samples, 135 upregulated and 144 downregulated transcripts were identified (Supplementary Table 2). The smaller number of DETs in AsymAD indicated that AsymAD might have lower level of molecular pathological changes compared to AD. Some dysregulated transcripts were shared between AsymAD and AD, while some were specific in AsymAD or AD samples. Specifically, 1,470 transcripts were downregulated in AD but showed no change in AsymAD samples, and 975 transcripts showed no change in AsymAD but upregulated in AD samples (Figure 1E). There were 98 transcripts that were downregulated and 89 transcripts that were upregulated in AsymAD but exhibited no change in AD samples. Among these dysregulated transcripts, 43 were upregulated, whereas 41 were downregulated in both AsymAD and AD samples. Further enrichment analysis revealed that upregulated DETs in AsymAD samples were enriched in metabolism-related processes, such as “Regulation of steroid biosynthetic process,” “Regulation of lipid biosynthetic process,” and “Regulation of steroid metabolic process,” while downregulated DETs showed enrichment of localization and transport processes, such as “Protein localization to nucleus” and “Nuclear export” (Figure 1F). Upregulated DETs in AD samples showed enrichment in synaptic functions, such as “Regulation of trans-synaptic signaling,” “Modulation of chemical synaptic transmission,” and “Synapse organization,” whereas downregulated DETs were enriched in cellular response processes, such as “Cellular response to abiotic stimulus,” “Cellular response to environmental stimulus,” and “Cellular response to zinc ion” (Figure 1G). In addition, we compared the AsymAD and AD transcriptomes and identified 95 upregulated and 37 downregulated transcripts in AD samples (Supplementary Figure 1A, Supplementary Table 3). The upregulated transcripts were enriched in “Positive regulation of cell-matrix adhesion,” “Cell cycle arrest,” and “Iron-ion transport,” while downregulated transcripts were enriched in “Negative regulation of cell-matrix adhesion,” “Negative regulation of cysteine-type endopeptidase activity involved in apoptotic process,” and “Negative regulation of blood vessel endothelial cell migration” (Supplementary Figure 1B). In summary, we characterized AsymAD and AD transcriptome and its dysregulation at transcript resolution.

### Transcripts showed inconsistent expression pattern with host genes

**FIGURE 2:**
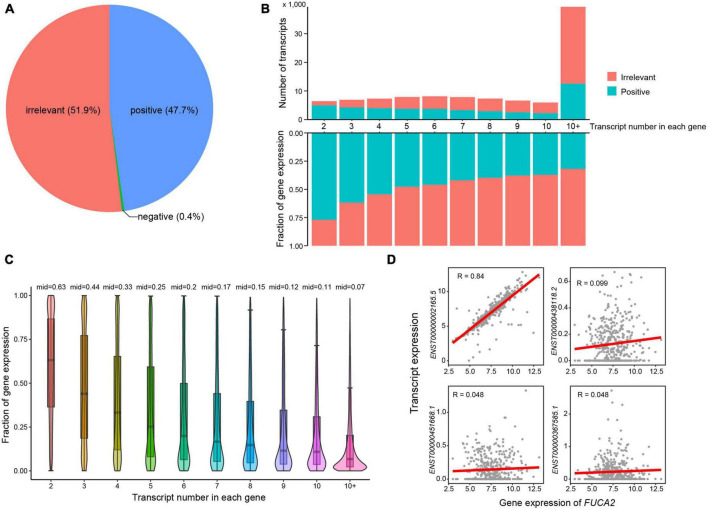
Expression correlations between transcripts and host genes. **(A)** Pie chart showing the percentages of transcripts that were positively and negatively correlated or irrelevant with host genes. **(B)** The number and expression fractions of positively correlated or irrelevant transcripts. **(C)** The expression fractions of positively correlated transcripts in host genes. **(D)** Expression correlations between *FUCA2* gene expression and its transcripts.

To investigate whether most transcripts showed consistent expression patterns with their host genes, we next calculated expression correlations between transcripts and the corresponding host genes (see Methods). Approximately half of all transcripts showed irrelevant expression correlation with their host genes (Figure 2A). As expected, the number of irrelevant transcripts increased with larger number of transcripts in single genes (Figure 2B). These observations highlighted the importance of transcript-level investigation of AD transcriptome. In addition, the expression fractions of positively correlated transcripts decreased with the increasing numbers of transcripts in single genes. More specifically, the median expression fraction of positively correlated transcripts was 0.63 in genes that had two transcripts (Figure 2C), while the median values of expression fractions were all below 0.5 in genes that expressed more than two transcripts. For example, the gene *FUCA2* expressed four transcripts, i.e., *ENST00000002165.5*, *ENST00000438118.2*, *ENST00000451668.1*, and *ENST00000367585.1* (Figure 2D). Transcript *ENST00000002165.5* showed high expression correlation with host gene *FUCA2*, while neither of the other three transcripts exhibited any expression correlation. Conclusively, our results highlighted that transcript-level analysis revealed a higher resolution of AD transcriptome than those in gene level.

### Abnormal splicing is frequent during the progression of AD

**FIGURE 3:**
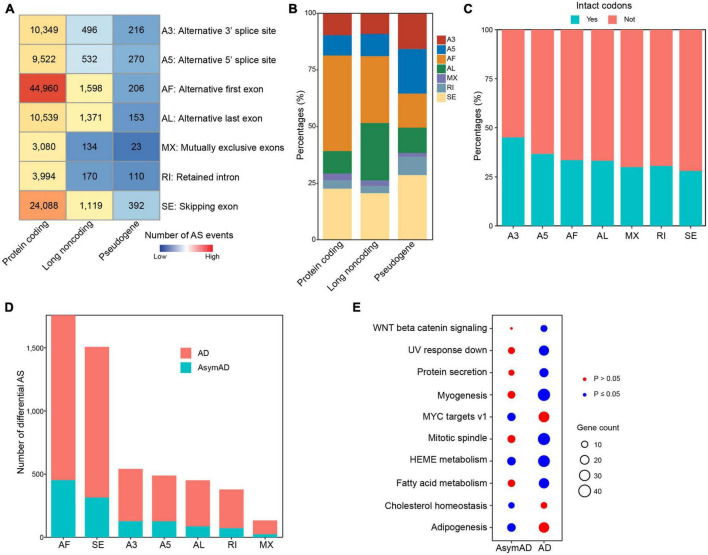
Alternative splicing events in Alzheimer’s disease (AD) samples. **(A)** Heatmap showing the number of different alternative splicing event (ASE) types in all samples, including non-demented control, asymptomatic AD (AsymAD), and AD samples. **(B)** Bar plots showing the percentages of different ASE types in protein coding genes, long non-coding genes, and pseudogenes. **(C)** Bar plots showing the percentages of intact codons in different ASE types. **(D)** The number of differentially spliced alternative splicing events (ASEs) in AsymAD and AD samples. **(E)** Enriched pathways by differentially spliced ASEs in AsymAD and AD samples. Red dots indicate *p* > 0.05, and blue dots indicate *p* ≤ 0.05.

Alternative splicing is supposed to make a major contribution to transcriptional variations in human diseases. We next identified ASEs in all samples, including seven different ASE types, i.e., alternative 3’ splice site (A3), alternative 5’ splice site (A5), alternative first exon (AF), alternative last exon (AL), mutually exclusive exons (MX), retained intron (RI), and skipping exon (SE). As expected, we identified the most ASEs in protein coding genes (Figure 3A). AF was the most frequent (*n* = 44,960, 42.2%) ASE type in protein coding genes, followed by SE (*n* = 24,088, 22.6%) (Figure 3B). In long non-coding RNA (lncRNA) genes, AF (*n* = 1,598, 29.5%) and AL (*n* = 1,371, 25.3%) were the most frequent ASE types. In pseudogenes, SE was the ASE type with the largest number. We next examined whether sequences affected by ASEs had intact codons. In all ASE types, over half sequences did not have intact codons, indicating that most ASEs might cause frame shift (Figure 3C). We also examined these in separate sample groups and found that the number distribution of different ASE types (Supplementary Figure 2), percentages of different ASE types (Supplementary Figure 3), and intact codons (Supplementary Figure 4) showed no notable differences in non-demented control, AsymAD, and AD sample groups. Comparing with non-demented control samples, AD samples showed more differentially spliced ASEs than AsymAD (Figure 3D). In total, 4,056 and 1,200 differentially spliced ASEs were identified in AD and AsymAD samples, respectively (Supplementary Tables 4, 5). AF and SE events had the largest number of differentially spliced events in both AsymAD and AD samples. Differentially spliced genes (DSGs) in AsymAD were enriched in “Adipogenesis,” “HEME metabolism,” and “MYC targets,” while DSGs in AD showed enrichment in “Myogenesis,” “Mitotic spindle,” and “Fatty acid metabolism” (Figure 3E). These results revealed the differences of alternative splicing variations between AsymAD and AD.

### Isoform switching events in AsymAD and AD

**FIGURE 4:**
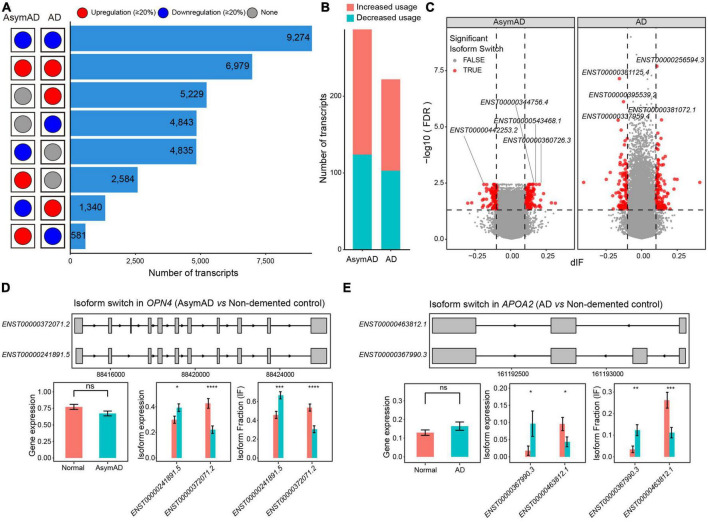
Isoform switching events in Alzheimer’s disease (AD). **(A)** The number of transcripts that showed different dysregulation patterns in asymptomatic AD (AsymAD) and AD samples. **(B)** The number of transcripts that showed increased or decreased usage in AsymAD or AD samples. **(C)** Volcano plots showing the difference of switching isoforms in AsymAD and AD samples. **(D)** Isoform switch in the *OPN4* gene (AsymAD vs. non-demented control). **(E)** Isoform switch in the *APOA2* gene (AD vs. non-demented control). *p ≤ 0.05, **p ≤ 0.01, ***p ≤ 0.001, and ****p ≤ 0.0001.

To further investigate the transcript variations in AsymAD and AD, we examined transcripts that showed greater than 20% changes compared to the non-demented control samples. A large number of transcripts showed consistent expression changes (>20% higher or lower) in both AsymAD and AD samples (Figure 4A). There were 5,229 upregulated and 4,843 downregulated transcripts that showed expression changes in only AD samples, whereas 4,843 transcripts were downregulated and 2,584 transcripts were upregulated in only AsymAD samples. In addition, 1,340 transcripts showed downregulated expression in AsymAD but upregulated expression in AD, while 581 transcripts were upregulated in AsymAD but downregulated in AD. Next, we identified isoform switching events in AsymAD and AD samples, respectively (see Methods). A total of 163 and 119 transcripts showed increased usage, while 124 and 103 transcripts exhibited decreased usage in AsymAD and AD, respectively (Figures 4B, C and Supplementary Table 6). We performed enrichment analysis of dysregulated genes that showed isoform switching events. Those genes that had isoform switching events in AsymAD samples were enriched in biological processes of visual system, such as “Eye development,” “Visual system development,” and “Retinal rod cell development” (Supplementary Figure 5A). In AD samples, genes that had isoform switching events were enriched in regulation of synaptic functions, such as “Modulation of chemical synaptic transmission,” “Regulation of trans-synaptic signaling,” and “Regulation of postsynaptic membrane potential” (Supplementary Figure 5B). For example, in the *OPN4* gene, transcript *ENST00000372071.2* had one more exon than transcript *ENST00000241891.5* (Figure 4D). The *OPN4* gene, a photosensitive protein, not only participates in the non-visual imaging system of animals (Tian and Copenhagen, 2003), but also in the regulation of circadian rhythm (Van Gelder, 2001). Circadian rhythm dysfunction and sleep disturbances are associated with aging and neurodegenerative diseases, including mild cognitive impairment (MCI) and AD. The *OPN4* gene has been reported to be associated with AD (Bacalini et al., 2022), which showed no expression changes between AsymAD and non-demented control samples. Transcript *ENST00000241891.5* showed significant upregulation, while transcript *ENST00000372071.2* was significantly downregulated in AsymAD samples. Additionally, among transcripts expressed from gene *OPN4*, the usage of transcript *ENST00000241891.5* was significantly increased, while the usage of transcript *ENST00000372071.2* decreased in AsymAD. In the *APOA2* gene, transcript *ENST00000367990.3* expressed four exons, whereas transcript *ENST00000463812.1* had three exons (Figure 4E). Transcript *ENST00000367990.3* was upregulated, whereas transcript *ENST00000463812.1* showed downregulation in AD samples. Compared to non-demented control samples, gene *APOA2* expressed higher proportion of transcript *ENST00000367990.3* in AD. The regulation and metabolism of lipid have been demonstrated to play important roles in the disease progression of AD (Yin, 2023). The *APOA2* gene belongs to the apoprotein gene family, wherein *APOE* has been demonstrated to play roles in AD. The switch between transcript *ENST00000367990.3* and *ENST00000463812.1* indicates the potential regulatory role of the *APOA2* gene in the progression of AD. In addition, we examined all isoform switching events in previously reported AD-related risk genes (Keren-Shaul et al., 2017; Grubman et al., 2019; Leng et al., 2021; Mertens et al., 2021; Bellenguez et al., 2022), and found 34 risk genes have at least one isoform switching event. For example, the *APP* gene showed switch between transcript *ENST00000348990.5* and *ENST00000354192.3* (Supplementary Figure 6), which was reported to have AD-related risk loci in GWAS analysis (Bellenguez et al., 2022). In summary, these results revealed the transcriptional reprograming in AsymAD and AD.

### RBPs regulate transcript variations in AsymAD and AD

**FIGURE 5:**
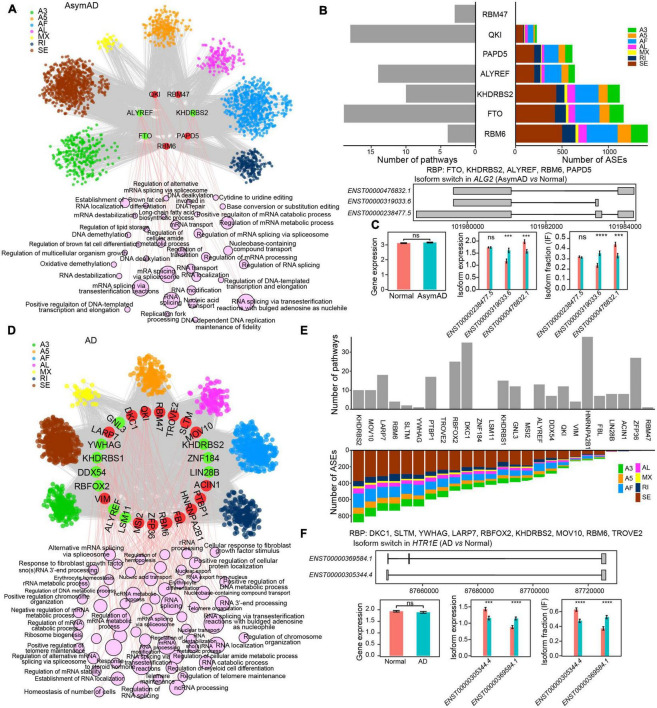
RNA binding protein (RBP) regulatory network in Alzheimer’s disease (AD). **(A)** RBP-alternative splicing event (ASE) regulatory network in asymptomatic AD (AsymAD) samples. **(B)** Bar plots showing the number of regulated ASEs and enriched pathways in AsymAD samples. **(C)** Isoform switch in the *ALG2* gene in AsymAD samples. **(D)** RBP-ASE regulatory network in AD samples. **(E)** Bar plots showing the number of regulated ASEs and enriched pathways in AD samples. **(F)** Isoform switch in the *HTR1E* gene in AD samples. ***p ≤ 0.001 and ****p ≤ 0.0001.

To further examine the regulatory role of RBPs, we built RBP-ASE regulatory networks in AsymAD and AD, respectively (see Methods). In the RBP-ASE network of AsymAD, four upregulated (QKI, RBM47, RBM6, and PAPD5) and three downregulated (KHDRBS2, ALYREF, and FTO) RBPs modulated 5,190 ASE events (Figure 5A and Supplementary Table 7). RBM6 regulated the largest number of ASEs, while FTO regulated the most pathways (Figure 5B). For example, gene *ALG2* was predicted to be regulated by five RBPs (FTO, KHDRBS2, ALYREF, RBM6, and PAPD5) in AsymAD, which expressed three transcripts, i.e., *ENST00000476832.1*, *ENST00000319033.6*, and *ENST00000238477.5* (Figure 5C). Transcript *ENST00000319033.6* showed upregulation and increased usage, while transcript *ENST00000476832.1* exhibited downregulation and decreased usage in AsymAD. In the RBP-ASE regulatory network of AD, 15 upregulated (LARP7, DKC1, QKI, RBM47, TROVE2, SLTM, MOV10, ACIN1, PTBP1, HNRNPA2B1, FBL, RBM6, ZFP36, MSI2, and VIM) and 10 downregulated (GNL3, KHDRBS2, ZNF184, LIN28B, LSM11, ALYREF, RBFOX2, DDX54, KHDRBS1, and YWHAG) RBPs modulated 9,918 ASE events (Figure 5D and Supplementary Table 8). KHDRBS2 regulated the largest number of ASEs in AD, whereas HNRNPA2B1 regulated the most pathways that were enriched by differentially spliced ASEs (Figure 5E). For instance, multiple RBPs were predicted to regulate gene *HTR1E* in AD (Figure 5F). In gene *HTR1E*, transcript *ENST00000369584.1* expressed one more exon than transcript *ENST00000305344.4*. The expression level and usage of transcript *ENST00000369584.1* significantly increased, while those of transcript *ENST00000305344.4* decreased in AD.

## Discussion

Differentially spliced AS events is implicated in the progression of AD (Raj et al., 2018; Han et al., 2020; Zafarullah et al., 2023). In this study, we investigated transcriptome changes throughout the progression of AD pathogenesis at transcript level. By analyzing transcript and ASE patterns at different stages of AD progression, we aimed to identify transcript or splicing events that may be associated with specific stages of the disease and could serve as potential biomarkers for early diagnosis or disease monitoring. We compared DETs between AsymAD and AD, and found that there were both shared and specific changes in transcript levels during the progression of AD. These results suggested that there were different patterns of transcript expression that were specific to different stages of the disease, these specific transcripts could be potential markers for diagnosis of AD progression. Interestingly, we observed that 581 transcripts were upregulated in AsymAD but downregulated in AD samples compared to the non-demented control. These transcripts might be specific biomarkers for AsymAD, or be able to prevent or slow down the progression from moderate AD to advanced AD. Much more computational and experimental work is needed to explore this valuable observation, which points a very good direction for further research of AD. The number of DETs in AsymAD was much lower than that in AD, which was consistent with previous studies (Patel et al., 2019; Fan et al., 2021). As the early stage of AD, AsymAD might have lower level of molecular pathological changes, thus showed much smaller number of differentially expressed transcripts and genes.

Our analysis identified a total of 113,322 ASEs, over 90% of which were observed in protein-coding genes. The most frequently observed ASE type in protein-coding genes was AF and SE events. We found that AF events had the highest number of differentially spliced ASEs in AsymAD and AD, suggesting that alterations in ASEs, particularly AF, might play a crucial role in the pathogenesis of AD. Isoform switching, which can have complex and multifaceted effects on RNA splicing, was also observed in AsymAD and AD. We identified 287 significant isoform switching events in the AsymAD samples and 222 in the AD samples. For example, we observed no difference in the gene-level expression of gene *OPN4*, but the expression and usage of its two transcripts exhibited opposite changes. These findings suggested that isoform switch might be a potential mechanism underlying AD pathogenesis, and further investigation is needed to fully understand its role in disease progression.

RBPs play a critical role in modulating RNA splicing (Fu and Ares, 2014; Marasco and Kornblihtt, 2022). In this study, we identified 7 and 25 differential RBPs in AsymAD and AD samples, respectively. We constructed RBP-ASE regulatory networks that represented valuable resources for further investigation of alternative splicing dysregulation in the progression of AD. We anticipate that many of the relationships identified in this study will be confirmed with the emergence of improved biotechnology for detecting RBP binding and the increasing volume of publicly available resources. These networks will provide insights into the molecular mechanisms underlying AD pathogenesis and may lead to the development of new therapeutic strategies targeting RBPs. To construct the RBP-ASE regulatory network, we retrieved a list of RBPs and their binding data from the Encyclopedia of RNA Interactomes (ENCORI) database (Li et al., 2014). The Spearman correlations were calculated between each RBP and differentially spliced ASE. Combination of correlation analysis and CLIP binding evidence has been shown to be an appropriate computational strategy to construct high-confidence RBP-ASE regulatory networks (Li et al., 2019; Hu et al., 2022). Although further experimental validation is needed, our computation-centered study also makes a valuable contribution to the understanding and exploration of the transcriptional diversity involved in AD progression. Some RBPs identified by our analysis have been reported to be play roles in AD. For example, our analysis found that HNRNPA2B1 was upregulated in AD samples, which was consistent with the previous study (Kavanagh et al., 2022). HNRNPA2B1 has demonstrated to interact with tau protein in regulating the progression of AD, and reduction of HNRNPA2B1 could reduce the pathological formation of tau protein (Jiang et al., 2021). PTBP1 was found to repress the alternative isoform of CD33, which is related to AD risk (van Bergeijk et al., 2019).

An advantage of our study is that we investigated the progression of AD from moderate to severe stages, not just differences between AD and non-demented control samples. This study provided a comprehensive understanding of the molecular mechanisms underlying AD pathogenesis. Future studies should aim to experimentally validate the relationships between alternative splicing events, isoform switching, RBPs in AD pathogenesis. These validations will confirm the reliability and significance of our findings and may lead to the development of new diagnostic and therapeutic strategies for AD. Single-cell studies have advanced the understanding of AD pathogenesis at single-cell level, and cell-type specific findings have revealed cellular contributions to AD progression (Mathys et al., 2019; Luquez et al., 2022). Although single-cell findings in alternative splicing would be valuable to the progression of AD, most current single-cell RNA-seq datasets are insufficient to identify alternative splicing and isoform switching events at single-cell level, which is more lacking in AD samples. Our bulk RNA-seq based study also make a valuable contribution to the understanding and exploration of the transcriptional diversity involved in AD progression. With the development of single-cell transcriptome technologies (Arzalluz-Luque and Conesa, 2018; Tian et al., 2021; Shi et al., 2023), we believe that we will have chance to analyze alternative splicing and isoform switching events at single-cell level in the near future.

In summary, our study provides a comprehensive analysis of transcriptional complexity and systematically characterizes the dysregulation of AS and its biological and clinical implications in the progression of AD. We identified significant isoform switching events and described the dysregulation of AS mediated by RBPs. The resources we provided here will aid understanding and exploring the transcriptional diversity in AD progression, and we anticipate that it will inspire fundamental studies and precision medicine in AD research.

## Data availability statement

The original contributions presented in this study are included in the article/Supplementary material, further inquiries can be directed to the corresponding authors.

## Author contributions

SL and RZ conceived and designed the project and supervised the project. HW performed the data analysis and visualization. JW assisted in data collection and interpreted results. XH assisted in data analysis. CZ interpreted results. JZ and PW assisted in data curation. SL wrote the manuscript with comments from all the other authors. All authors read and approved the final manuscript.

## Footnotes

1
https://www.synapse.org/#!Synapse:syn2580853

2
http://www.r-project.org
