# Supplementary material for: Comprehensive transcript-level analysis reveals transcriptional reprogramming during the progression of Alzheimer’s disease
Source: Front Aging Neurosci. 2023 Jun 15;15:1191680. doi: 10.3389/fnagi.2023.1191680 (PMC10308376; doi:10.3389/fnagi.2023.1191680)
Supplement: Supplementary file 9 [file Table_9.DOCX]

**Supplementary tables**

**Supplementary Table S1.** Differentially expressed transcripts in AD samples.

**Supplementary Table S2.** Differentially expressed transcripts in AsymAD samples.

**Supplementary Table S3.** Differentially expressed transcripts between AD and AsymAD samples.

**Supplementary Table S4.** Differential ASEs in AD.

**Supplementary Table S5.** Differential ASEs in AsymAD.

**Supplementary Table S6.** Isoform switching events in AsymAD and AD samples.

**Supplementary Table S7.** RBP-ASE regulatory network in AsymAD.

**Supplementary Table S8.** RBP-ASE regulatory network in AD.

**Supplementary figures**

**
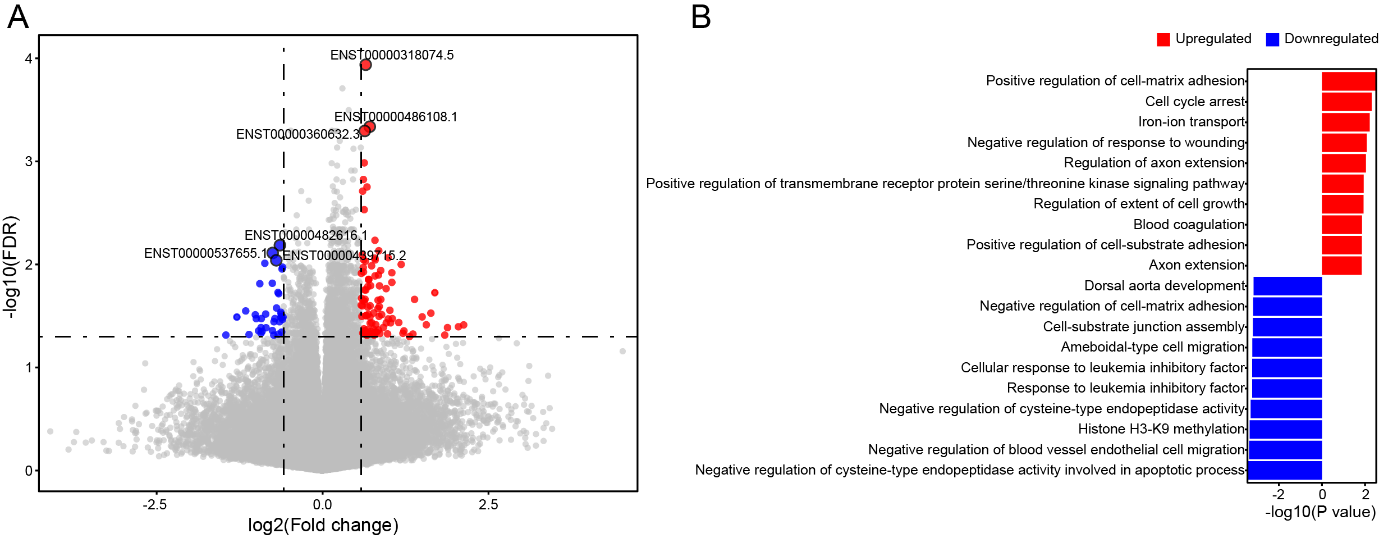
**

**Supplementary Figure S1. Comparison between AD and AsymAD samples.** (**A**) Volcano plot showing the differentially expressed transcripts between AD and AsymAD samples. (**B**) Bar plots showing the enriched biological processes by DETs between AD and AsymAD.

**
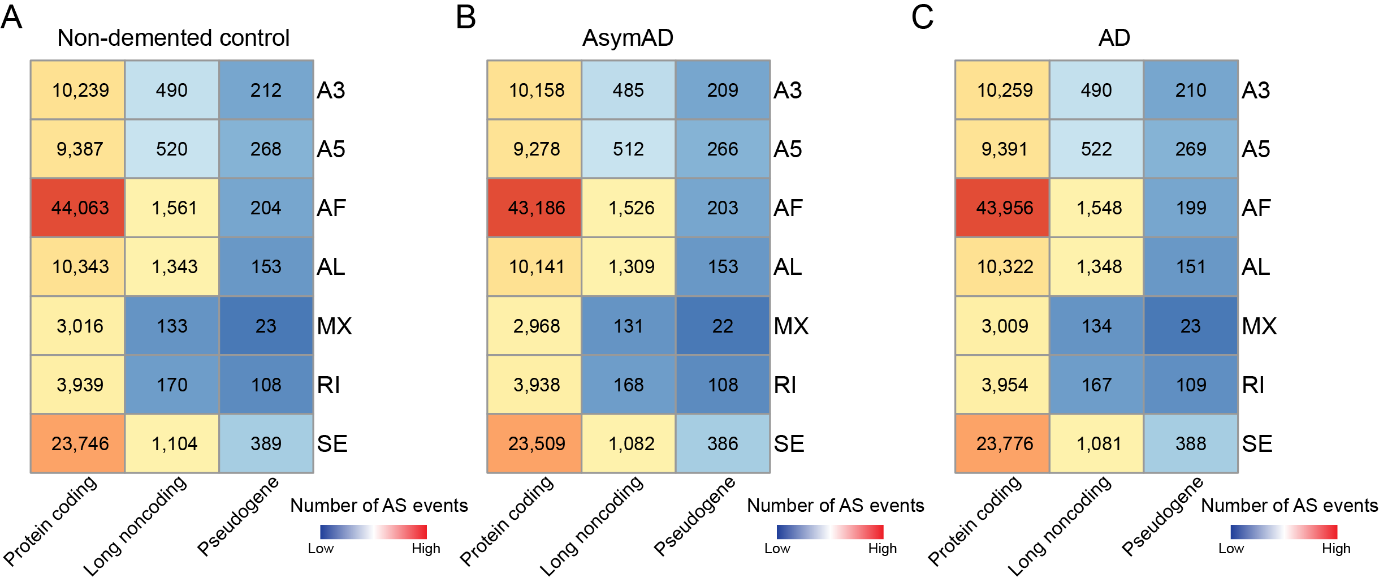
**

**Supplementary Figure S2. Heatmaps showing the number of different types of ASEs in different gene types in non-demented control** **(A) AsymAD (B), and AD samples (C).**

**
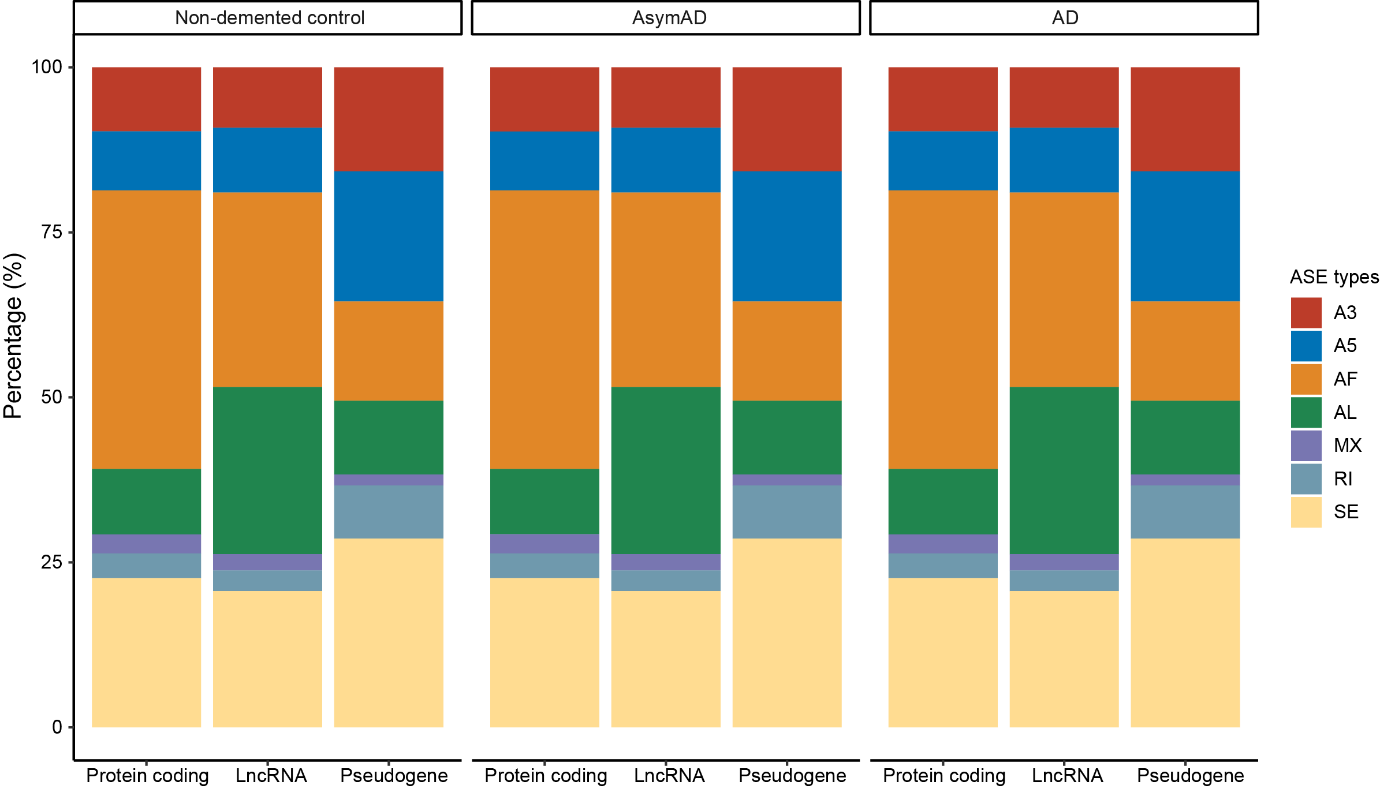
**

**Supplementary Figure S3. Bar plots showing the percentages of different ASE types in non-demented control, AsymAD, and AD samples, respectively.**

**
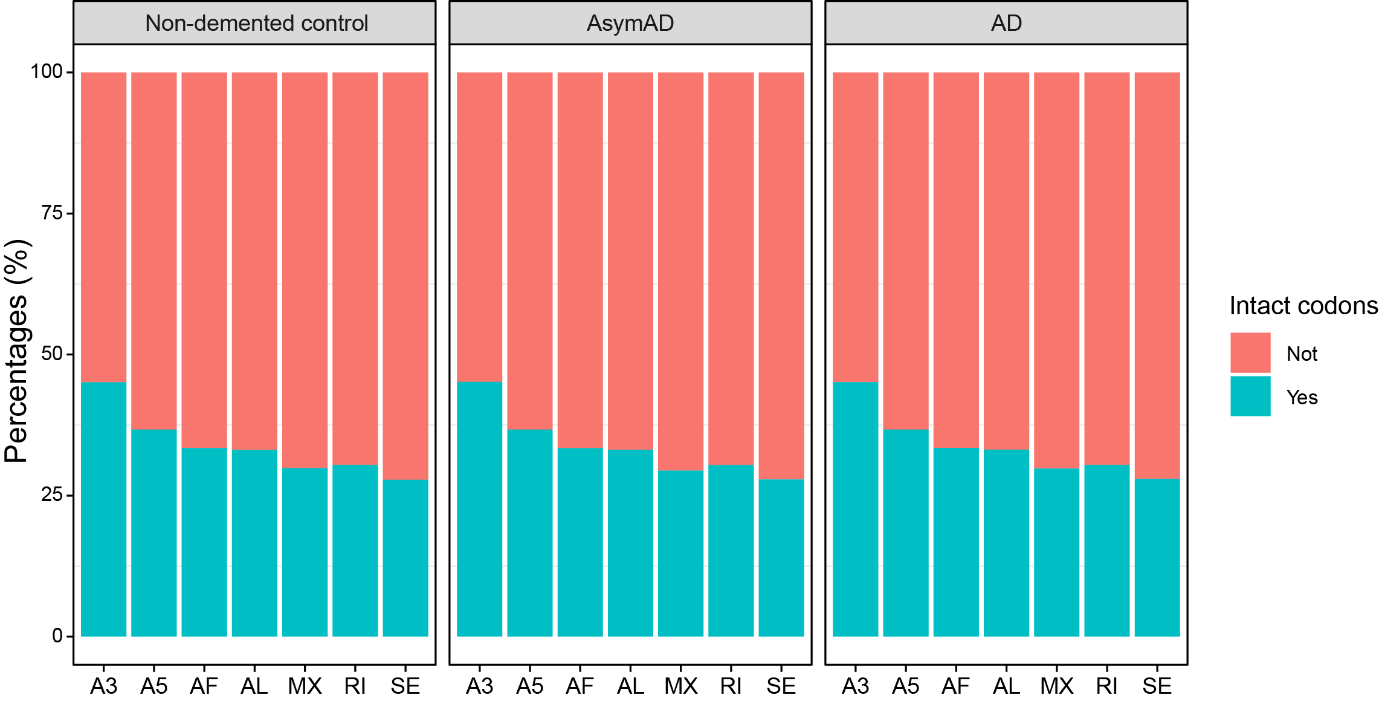
**

**Supplementary Figure S4. Bar plots showing the percentages of intact codons in different ASE types in non-demented control, AsymAD, and AD samples.**

**
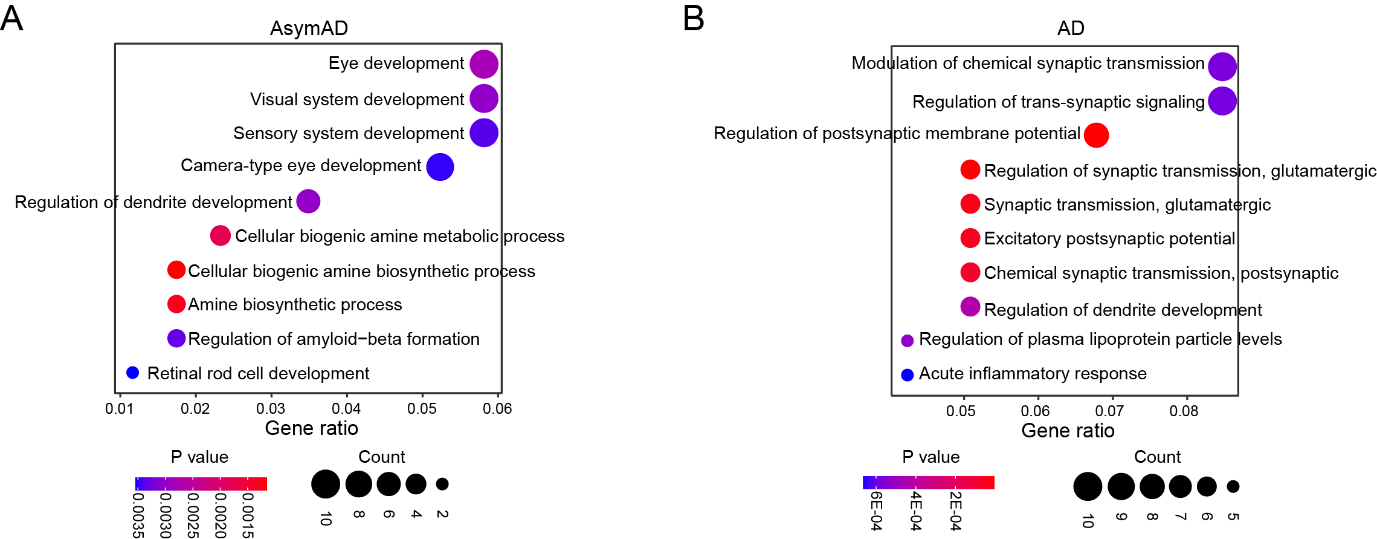
**

**Supplementary Figure S5. Functional enrichment analysis of genes that had isoform switching events in AsymAD (A) and AD samples (B).**

**
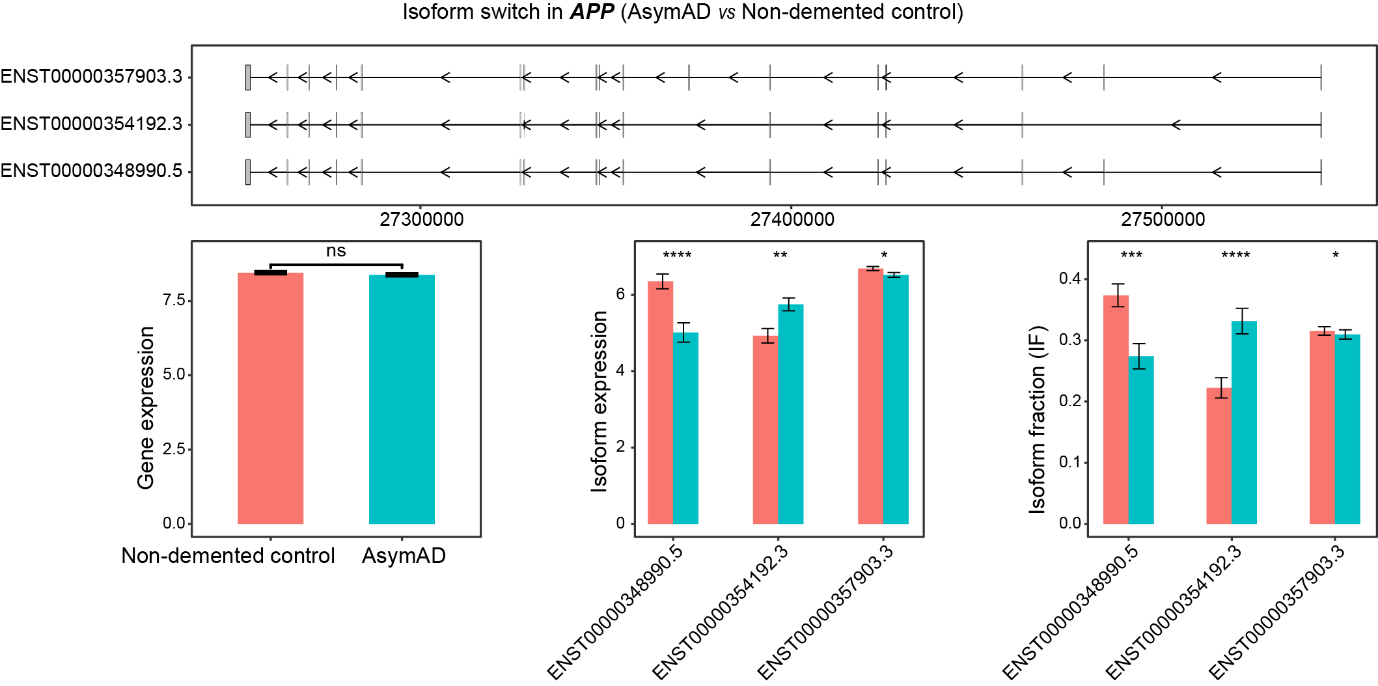
**

**Supplementary Figure S6. Isoform switch in *APP* gene (AsymAD *vs* non-demented control).**
